# Supplementary material for: A Gedunin-Type Limonoid, 7-Deacetoxy-7-Oxogedunin, from Andiroba (Carapa guianensis Aublet) Reduced Intracellular Triglyceride Content and Enhanced Autophagy in HepG2 Cells
Source: Int J Mol Sci. 2022 Oct 28;23(21):13141. doi: 10.3390/ijms232113141 (PMC9655357; doi:10.3390/ijms232113141)
Supplement: Supplementary file 1 [file ijms-23-13141-s001.zip › ijms-1973941-supplementary.pdf]

International Journal of
Molecular Sciences

MDPI

SupplementaryMaterials

A Gedunin-Type Limonoid, 7-Deacetoxy-7-oxogedunin, from Andiroba (*Carapa guianensis* Aublet) Reduced Intracellular Triglyceride Content and Enhanced Autophagy in HepG2 Cells

Akifumi Nagatomo [1], Kiyofumi Ninomiya [1,2], Shinsuke Marumoto [3], Chie Sakai [1], Shuta Watanabe [1], Wakana Ishikawa [1], Yoshiaki Manse [1], Takashi Kikuchi [4,5], Takeshi Yamada [4], Reiko Tanaka [4], Osamu Muraoka [1,6] and Toshio Morikawa [1,6,*]

[1] Pharmaceutical Research and Technology Institute, Kindai University, 3-4-1 Kowakae, Higashi-Osaka 577-8502, Osaka, Japan
[2] School of Pharmacy, Shujitsu University, 1-6-1 Nishigawara, Naka-ku, Okayama 703-8516, Okayama, Japan
[3] Joint Research Center, Kindai University, 3-4-1 Kowakae, Higashi-Osaka 577-8502, Osaka, Japan
[4] Faculty of Pharmacy, Osaka Medical and Pharmaceutical University, 4-20-1 Nasahara, Takatsuki 569-1094, Osaka, Japan
[5] Faculty of Pharmacy, Toho University, 2-2-1 Miyama, Funabashi 274-8510, Chiba, Japan
[6] Antiaging Center, Kindai University, 3-4-1 Kowakae, Higashi-Osaka 577-8502, Osaka, Japan
* Correspondence: morikawa@kindai.ac.jp; Tel.: +81-6-4307-4306; Fax: +81-6-6729-3577

Table of Contents

Figure S1. Unedited blots for Figure 4B S2
Figure S2. Unedited blots for Figure 5A S3
Figure S3. ^{1}H-NMR (400 MHz, $CDCl_3$) spectrum of DAOG (**1**) S4
Figure S4. ^{13}C-NMR (100 MHz, $CDCl_3$) spectrum of DAOG (**1**) S5
Table S1. Numerical data of MTT assay for Figure 3 .... S6
Table S2. Numerical data of intracellular TG reduction assay for Figure 4A S6
Table S3. Numerical data of western blot analysis for Figure 4C S6
Table S4. Numerical data of western blot analysis for Figure 5B S7
Table S5. Numerical data of western blot analysis for Figure 5C S7
Table S6. Numerical data of mRNA expressions for Figure 5D S7

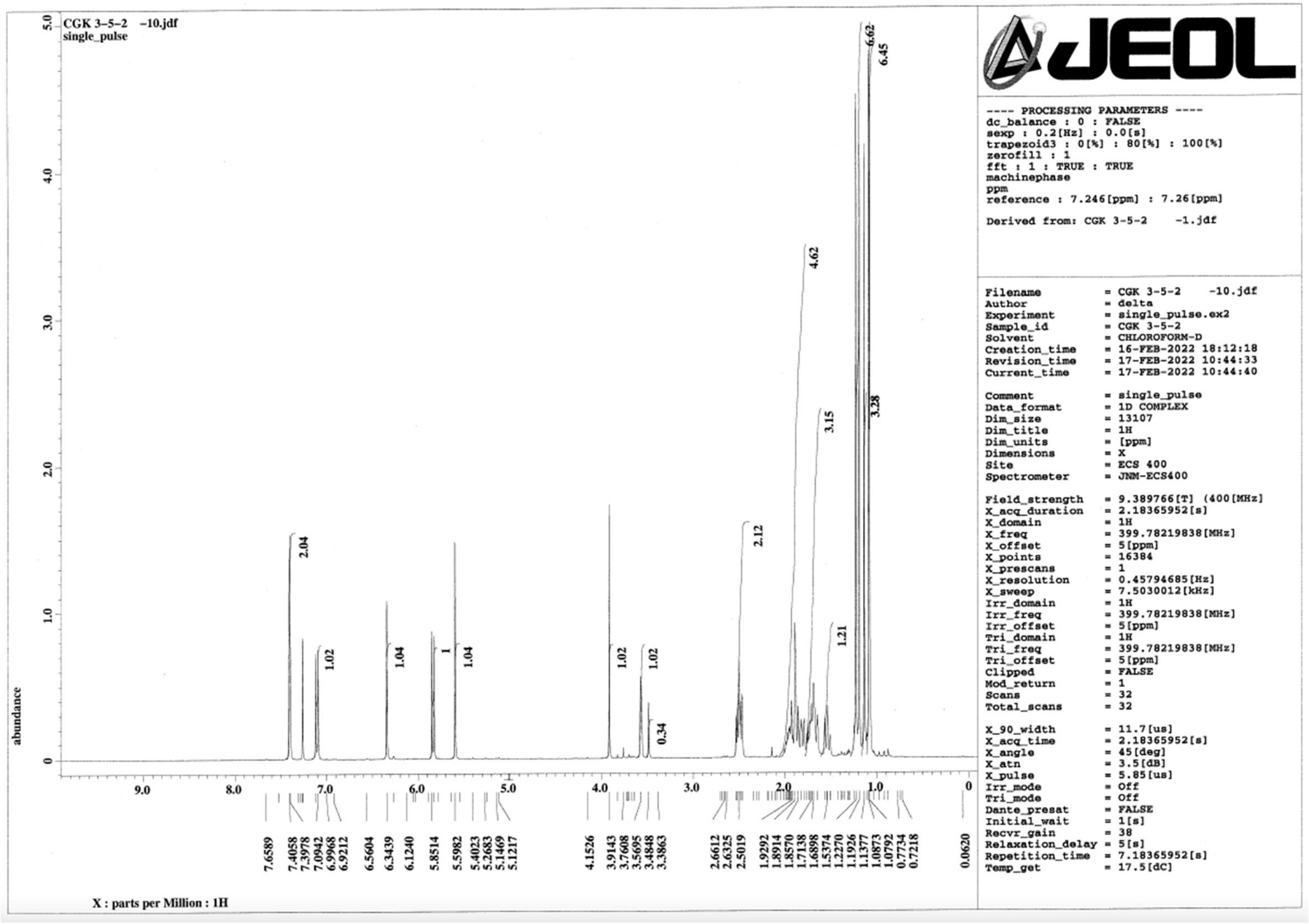

Figure S1. ^{1}H-NMR (400 MHz, $CDCl_3$) spectrum of DAOG (**1**).

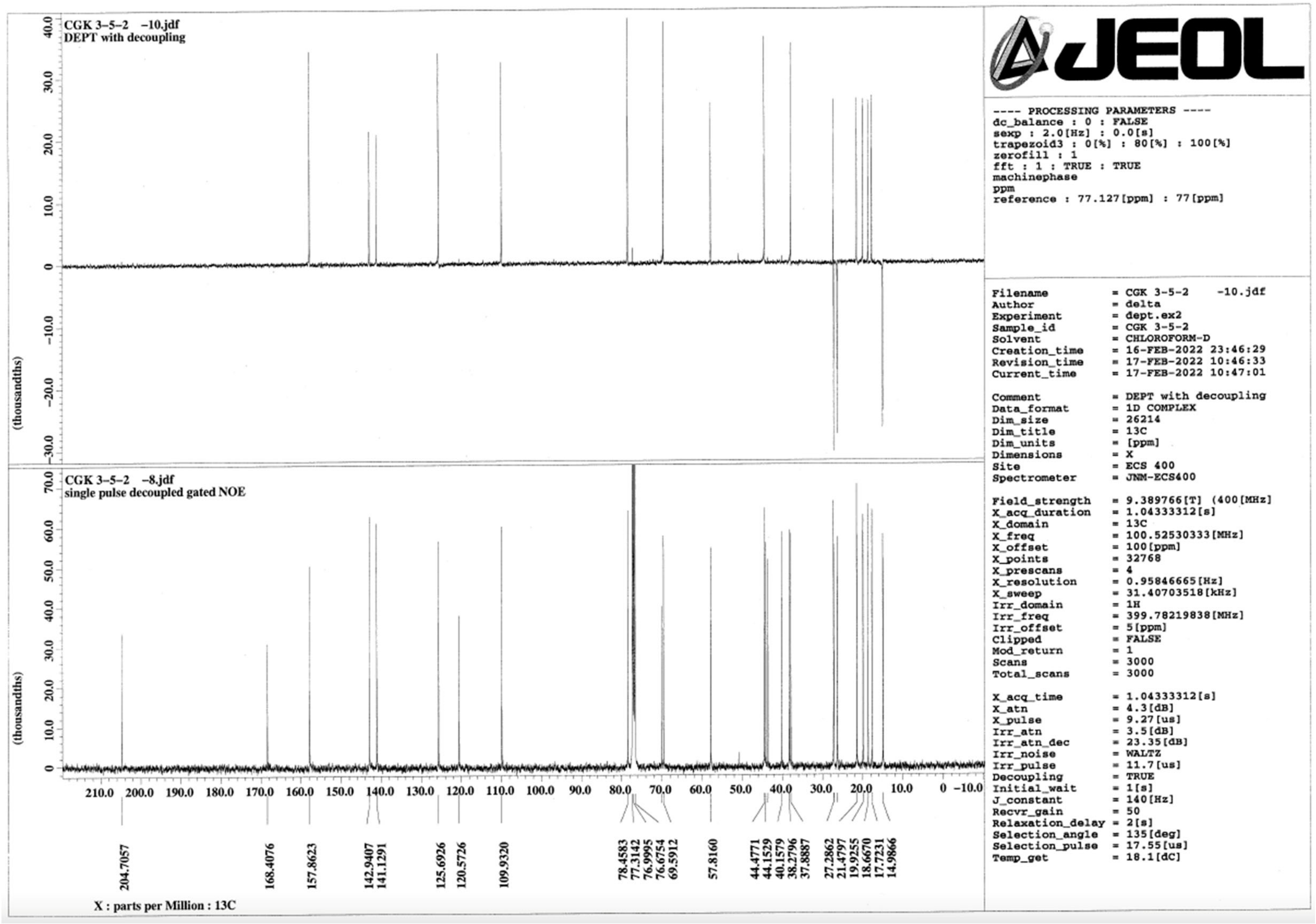

Figure S2. ^{13}C-NMR (100 MHz, $CDCl_3$) spectrum of DAOG (**1**).

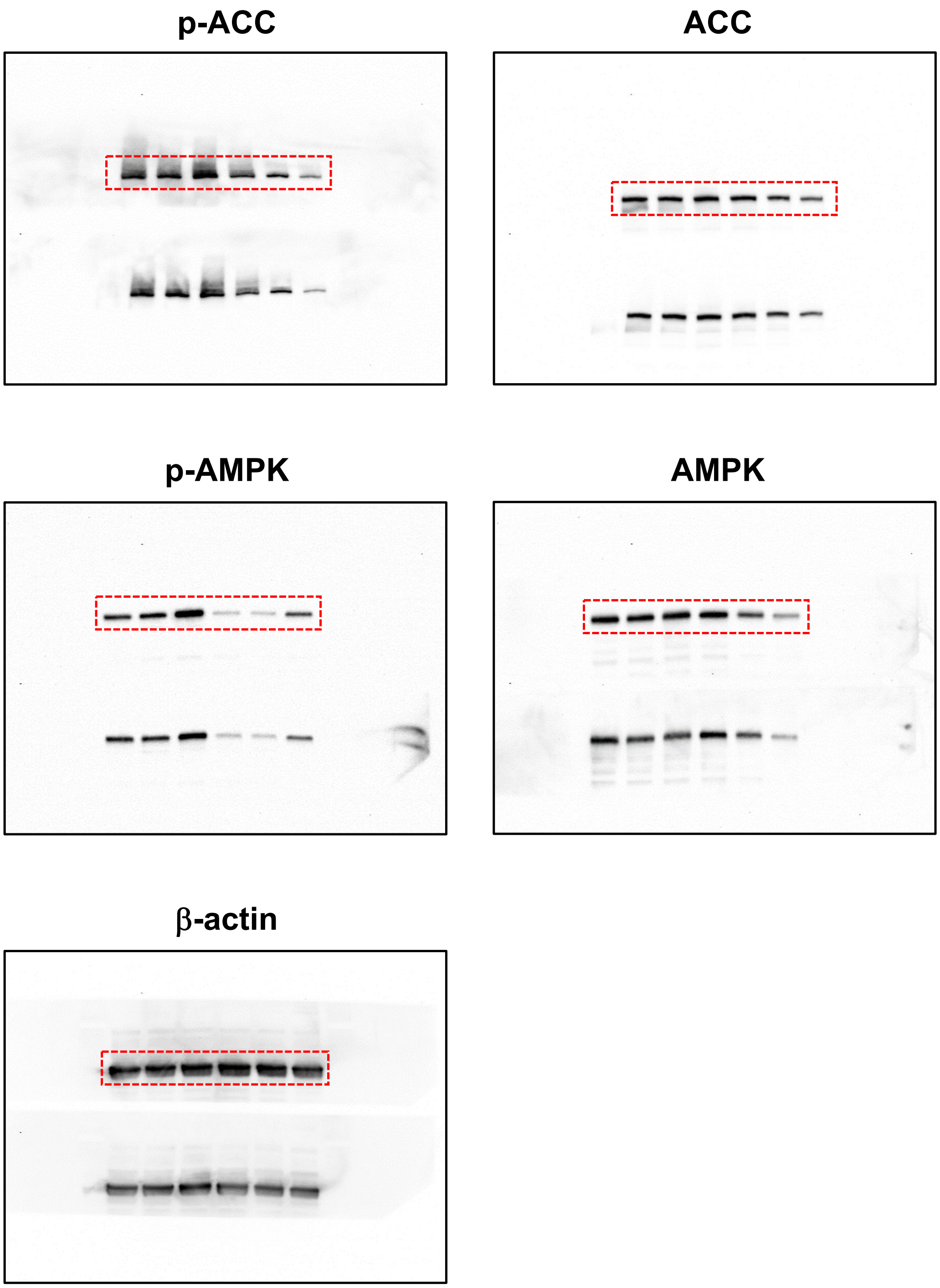

Figure S3. Unedited blots for Figure 4B.

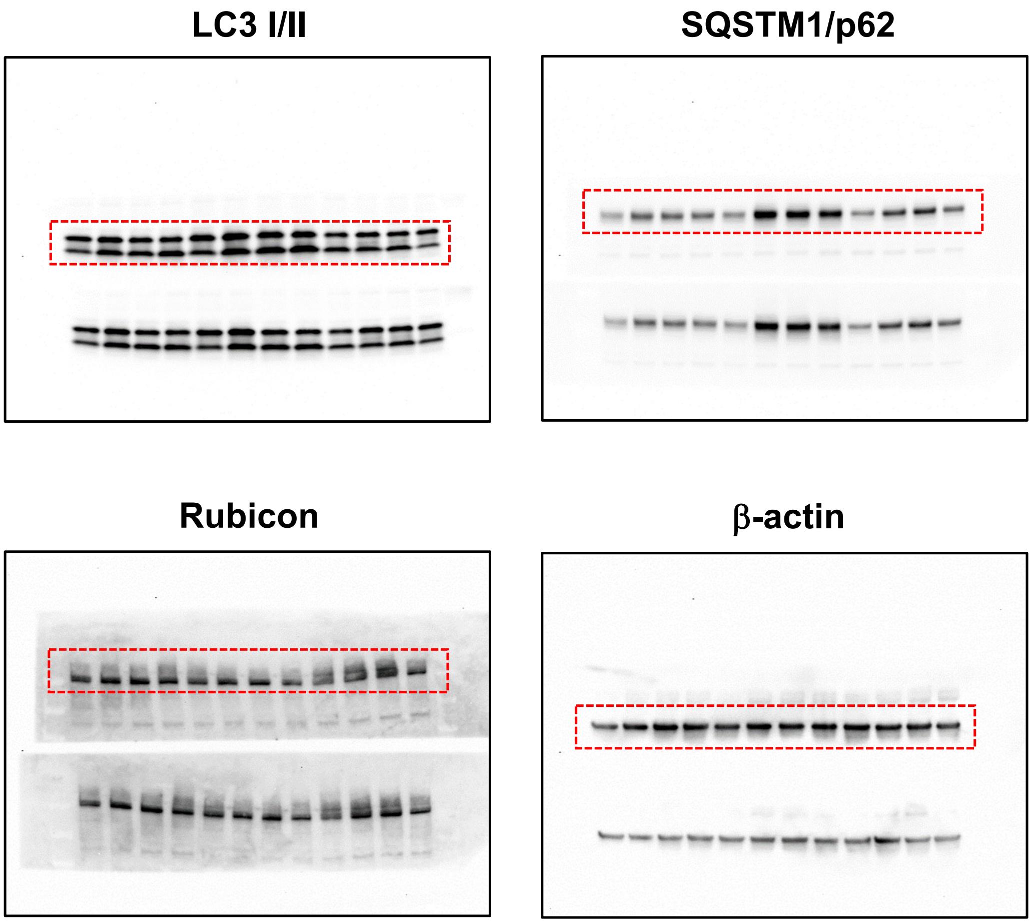

Figure S4. Unedited blots for Figure 5A.

Tabe S1. Numerical data of MTT assay for Figure 3.

Treatment	Cell viability (%)					
	**0 μM**	**2.5 μM**	**5 μM**	**10 μM**	**20 μM**	**40 μM**
DAOG (**1**)	100.0 ± 1.4	97.8 ± 0.6	98.0 ± 0.1	96.2 ± 0.5**	95.6 ± 0.2**	94.5 ± 0.7**
	**0 μM**	**1 μM**	**3 μM**	**10 μM**	**30 μM**	**100 μM**
BBR	100.0 ± 0.5	100.0 ± 0.5	100.0 ± 0.6	98.0 ± 0.2	96.2 ± 0.4**	1.6 ± 0.7**

Each value represents the mean ± S.E.M. (n = 4). Significantly different from the control, ** p <0.01 (Dunnett). DAOG; 7-deacetoxy-7-oxogedunin, BBR; berberine chloride.

Tabe S2. Numerical data of intracellular TG reduction assay for Figure 4A.

Treatment	Compound C	TG/protein in the homogenate (% of control)			
	**20 μM**	**0 μM**	**5 μM**	**10 μM**	**20 μM**
DAOG (**1**)	−	100.0 ± 1.7	91.2 ± 1.7*	80.1 ± 0.9**	71.3 ± 1.6**
	+	100.0 ± 2.0	90.5 ± 1.3**	86.3 ± 1.2**##	97.9 ± 0.9##
		**0 μM**	**7.5 μM**	**15 μM**	**30 μM**
BBR	−	100.0 ± 3.8	89.3 ± 3.4	91.2 ± 2.8	82.6 ± 2.4**
	+	100.0 ± 1.1	109.6 ± 1.8*##	107.3 ± 3.1##	149.0 ± 2.6**##

Each value represents the mean ± S.E.M. (n = 4). * p <0.05, ** p <0.01 *vs.* control cells treated with vehicle (Dunnett); ## p <0.01 *vs.* compound C-nontreated cells (Student's t). DAOG; 7-deacetoxy-7-oxogedunin, BBR; berberine chloride.

Tabe S3. Numerical data of western blot analysis for Figure 4C.

Protein	Compound C	Relative expression (fold increase)		
	**20 μM**	**Control**	**DAOG (1, 20 μM)**	**BBR (30 μM)**
p-AMPK/AMPK	−	1.00 ± 0.00	1.43 ± 0.07	3.21 ± 0.66**
	+	0.26 ± 0.04##	0.41 ± 0.06##	1.68 ± 0.47**
p-ACC/ACC	−	1.00 ± 0.00	0.86 ± 0.09	0.96 ± 0.14
	+	0.57 ± 0.09##	0.57 ± 0.04#	0.37 ± 0.06##

Each value represents the mean ± S.E.M. (n = 4). ** p <0.01 *vs.* control cells treated with vehicle (Dunnett); # p <0.05, ## p <0.01 *vs.* compound C-nontreated cells (Student's t). DAOG; 7-deacetoxy-7-oxogedunin, BBR; berberine chloride.

Tabe S4. Numerical data of western blot analysis for Figure 5B.

Protein	Treatment	Relative expression (fold increase)			
		**0 h**	**6 h**	**12 h**	**24 h**
LC3-II / β-actin	Control	1.00 ± 0.00	1.25 ± 0.10	0.93 ± 0.08	1.13 ± 0.08
	DAOG (**1**, 20 μM)	1.00 ± 0.00	1.43 ± 0.12	1.48 ± 0.18#	1.55 ± 0.17*
	BBR (30 μM)	1.00 ± 0.00	0.90 ± 0.15	1.13 ± 0.15	0.66 ± 0.18
SQSTM1/p62 / β-actin	Control	1.00 ± 0.00	1.60 ± 0.18*	1.24 ± 0.15	1.30 ± 0.12
	DAOG (**1**, 20 μM)	1.00 ± 0.00	2.74 ± 0.15**##	2.70 ± 0.22**##	2.18 ± 0.17**##
	BBR (30 μM)	1.00 ± 0.00	1.67 ± 0.19	2.06 ± 0.31**	1.28 ± 0.20
Rubicon / β-actin	Control	1.00 ± 0.00	1.08 ± 0.11	1.08 ± 0.12	1.12 ± 0.10
	DAOG (**1**, 20 μM)	1.00 ± 0.00	0.75 ± 0.03**	0.76 ± 0.03**	0.62 ± 0.08**#
	BBR (30 μM)	1.00 ± 0.00	1.03 ± 0.22	1.18 ± 0.30	0.80 ± 0.19

Each value represents the mean ± S.E.M. (n = 4); * p <0.05, ** p <0.01 *vs.* initial (0 h) expression (Dunnett); # p <0.05, ## p <0.01 *vs.* control group at each sampling point (Dunnett). DAOG; 7-deacetoxy-7-oxogedunin, BBR; berberine chloride.

Tabe S5. Numerical data of autophagy flux assay for Figure 5C.

Protein	CQ 100 μM	Relative expression (fold increase)		
		**Control**	**DAOG (1, 20 μM)**	**BBR (30 μM)**
LC3-II / β-actin	−	1.01 ± 0.06^{ab}	1.34 ± 0.06^{ac}	0.72 ± 0.04^{b}
	+	1.84 ± 0.05^{c}	2.58 ± 0.21^{d}	1.77 ± 0.21^{c}

Each value represents the mean ± S.E.M. (n = 4). Different letters indicate significant differences, p < 0.05 (Tukey-Kramer’s HSD). CQ; chloroquine, DAOG; 7-deacetoxy-7-oxogedunin, BBR; berberine chloride.

Tabe S6. Numerical data of mRNA expressions for Figure 5D.

Treatment	mRNA level	
	SQSTM1 / RPLP0	***RUBCN / RPLP0***
Control	1.02 ± 0.13	1.01 ± 0.08
DAOG (**1**, 20 μM)	2.93 ± 0.07**	0.63 ± 0.02**

Each value represents the mean ± S.E.M. (n = 4). Signifiantly different from the control, ** p <0.01 (Student’s t). DAOG; 7-deacetoxy-7-oxogedunin, BBR; berberine chloride.
